# Precision medicine in orthopaedics: A review of current technologies and future directions

**DOI:** 10.1002/ksa.70168

**Published:** 2025-11-01

**Authors:** Patrick Sadoghi, Amir Koutp, Elmar Herbst, Giuseppe Milano, Volker Musahl, Michael T. Hirschmann

**Affiliations:** ^1^ Department of Orthopaedics and Trauma Medical University of Graz Graz Austria; ^2^ Alps Surgery Institute Clinique Generale Annecy Annecy France; ^3^ Department of Trauma, Hand and Reconstructive Surgery University Hospital Münster Münster Germany; ^4^ Department of Medical and Surgical Specialties, Radiological Sciences and Public Health University of Brescia Brescia Italy; ^5^ Department of Bone and Joint Surgery ASST Spedali Civili Brescia Italy; ^6^ Department of Orthopaedic Surgery University of Pittsburgh Pittsburgh Pennsylvania USA; ^7^ University Department of Orthopedic Surgery and Traumatology Kantonsspital Baselland Bruderholz Switzerland; ^8^ Department of Clinical Research, Research Group Michael T. Hirschmann, Regenerative Medicine & Biomechanics University of Basel Basel Switzerland

**Keywords:** AI, custom implants, patient‐specific alignment, personalised medicine, robotic surgery

## Abstract

**Purpose:**

To synthesise the paradigm shift towards precision medicine in orthopaedics, where individual anatomical, biomechanical, molecular and kinematic characteristics are integrated into clinical decision‐making. Unlike traditional approaches applying uniform protocols, this review outlines how precision orthopaedics aims to tailor surgical techniques, implant selection, component positioning and rehabilitation strategies to the unique profile of each patient, thereby improving outcomes and predictability.

**Methods:**

This narrative review synthesises current concepts and evidence supporting patient‐specific care. The methods discussed encompass a wide range of technological and biological innovations, including advanced imaging, robotic‐assisted surgery, artificial intelligence (AI), molecular diagnostics, functional assessment tools and personalised therapeutic platforms that are shaping modern orthopaedic practice across multiple subspecialties.

**Results:**

In total knee arthroplasty, personalised alignment restores native joint lines, while robotic systems execute plans with submillimetre accuracy, reducing alignment outliers and potentially improving functional outcomes. In total hip arthroplasty, spinopelvic analysis mitigates instability risk, a critical factor for patients with spinal stiffness. Intraoperative technologies like robotics, patient‐specific instruments and augmented reality improve the precision of implant placement and reduce radiation exposure in trauma. Beyond arthroplasty, AI accelerates early diagnosis of osteoarthritis, while molecular biomarkers (e.g., alpha‐defensin) offer >95% accuracy in diagnosing periprosthetic joint infection. Finally, AI‐guided digital platforms and motion tracking are used to deliver personalised rehabilitation protocols.

**Conclusion:**

Precision medicine encompasses a wide range of powerful tools, many of which are already in clinical use. However, their full and effective integration requires continued research, long‐term validation, cost‐effectiveness analyses and interdisciplinary collaboration. The future of orthopaedics is anchored in delivering the right intervention for the right patient at the right time, guided by robust, individualised data and sound clinical judgement.

**Level of Evidence:**

Level V.

**Abbreviations**
3D: three‐dimensional
AI: artificial intelligence
AR: augmented reality
CT: computed tomography
EBJIS: European Bone and Joint Infection Society
EOS: low‐dose biplanar X‐ray system enabling weight‐bearing, simultaneous orthogonal imaging and 3D reconstruction
FKP: functional knee phenotype
HLA‐B27: human leucocyte antigen b27
IL‐6: interleukin‐6
IMUs: inertial measur
KA: kinematic alignment
MRI: magnetic resonance imaging
MSIS: musculoskeletal infection society
NGS: next‐generation sequencing
OARSI: Osteoarthritis Research Society International
PJI: periprosthetic joint infection
PSI: patient‐specific instruments
SNPs: single nucleotide polymorphisms
THA: total hip arthroplasty
TKA: total knee arthroplasty

## INTRODUCTION

Precision medicine, also known as personalised medicine, is increasingly shaping the clinical and scientific landscape of orthopaedics. The core principle is to tailor diagnostics, surgical procedures and rehabilitation strategies to the individual characteristics or phenotypes of each patient [10, 21, 49]. This paradigm shift is driven by technological innovation, refined biomechanical understanding and growing evidence supporting patient‐specific approaches across multiple subspecialties [26].

In contrast to traditional one‐size‐fits‐all models, precision orthopaedics acknowledges anatomical variability, biomechanical nuance and molecular diversity in musculoskeletal pathology [19]. Whether addressing coronal plane alignment in knee arthroplasty [37], spinopelvic dynamics in hip surgery [45], or soft tissue balance in shoulder and spine procedures, modern orthopaedics is evolving toward personalised surgical targets informed by high‐resolution imaging, kinematic modelling and artificial intelligence (AI)–assisted analytics [42].

Robotic‐assisted systems and patient‐specific instrumentation (PSI) have enabled greater surgical precision, particularly in joint replacement, where deviations as small as a few millimetres may influence long‐term outcomes [24]. Similarly, gait analysis, wearable sensors and AI‐based rehabilitation platforms offer data‐driven pathways to assess function and tailor recovery strategies [14].

Importantly, advances in genomics and biomarker discovery now allow clinicians to detect periprosthetic joint infections, inflammatory arthropathies, or bone tumours with greater specificity and at an earlier stage [2]. This integrative model combining radiographic, biomechanical and molecular data marks a shift not only in treatment but also in how orthopaedic pathology is conceptualised and classified.

This review synthesises the applications of precision medicine by following the logical arc of the patient journey: from preoperative planning and diagnostics, through intraoperative execution, and into postoperative assessment and rehabilitation. We will examine specific technologies, their demonstrated benefits and limitations, and their clinical relevance in improving outcomes. By outlining where these tools are used—and why they are not yet ubiquitous—we aim to provide a balanced overview of the current state and future directions of precision orthopaedics.

### Preoperative planning and diagnostics

The foundation of precision orthopaedics is a meticulous preoperative plan that integrates advanced imaging, biomechanical principles and computational analysis. Using imaging modalities such as X‐ray, computed tomography (CT) or magnetic resonance imaging (MRI), these systems create detailed 3D virtual reconstructions of the patient's anatomy, establishing a precise surgical blueprint.

### Imaging, AI and diagnostic precision

Advances in diagnostic imaging and AI are significantly enhancing the precision of orthopaedic assessment. AI‐based radiological tools can support early detection of musculoskeletal disorders, automate complex classifications and generate predictive models [34]. In osteoarthritis, machine learning algorithms can identify subtle features of cartilage degradation or bone changes on radiographs or MRI scans, often before they are apparent to the human eye, enabling proactive intervention [44]. Deep learning models also reduce interobserver variability in grading systems like Kellgren–Lawrence, improving consistency in research and clinical practice [43]. Beyond diagnostics, AI models integrate radiographic and clinical data to predict postoperative risks, such as complications or implant failure, allowing for patient stratification and preoperative optimisation [14, 41].

### Patient‐specific surgical planning

High‐resolution imaging forms the basis for personalised surgical strategies in arthroplasty.

In total knee arthroplasty (TKA), this enables a shift from conventional mechanical alignment (MA) to personalised alignment philosophies like kinematic alignment (KA) or functional alignment (FA). These strategies aim to replicate an individual's native joint line and soft‐tissue balance, recognising that constitutional varus or valgus is not inherently pathological [18, 48]. This approach is supported by phenotyping systems like the functional knee phenotype (FKP) and coronal plane alignment of the knee (CPAK) classifications, which document the wide variability of native alignment shown in nonosteoarthritic knees and challenge the one‐size‐fits‐all MA target [17, 20, 22, 23, 37, 40]. The clinical goal is to restore more natural kinematics and potentially improve patient satisfaction.

In total hip arthroplasty (THA), planning has evolved to account for the dynamic spinopelvic relationship. Traditional acetabular cup placement in a static ‘safe zone’ fails to account for individuals with spinal stiffness (e.g., from lumbar fusion), who have altered pelvic tilt between sitting and standing, increasing dislocation risk [16, 45]. Precision planning incorporates standing/sitting radiographs or functional imaging (e.g., EOS) to assess individual spinopelvic mobility, allowing surgeons to select patient‐specific cup orientation targets that optimise stability throughout the range of motion [31, 36].

### Intraoperative execution: Translating plans into precision

Executing a personalised plan requires technologies that can translate digital models into precise surgical action. An overview of these technologies is presented in Table 1.

**Table 1:** Overview of intraoperative precision technologies in orthopaedics.

Technology	Principle	Key benefits	Key limitations	Primary clinical applications
Robotic‐assisted surgery	Preoperative computed tomography (CT)‐based plan executed via a robotic arm with real‐time tracking.	Submillimetre accuracy; dynamic ligament balancing; high reproducibility.	High initial cost; learning curve; longer initial operative times.	Total knee arthroplasty (TKA), total hip arthroplasty (THA), spine surgery.
Patient‐Specific Instrumentation (PSI)	Preoperative plan translated into 3D‐printed, single‐use cutting guides.	Improved accuracy versus conventional; reduced instrument trays; potentially shorter OR time.	Additional cost of guide; plan is static (no intraoperative adjustment); dependent on imaging quality.	TKA, total shoulder/ankle arthroplasty.
Augmented reality (AR)	Overlays digital models and guides onto the surgeon's real‐world view.	Enhanced 3D visualisation; potentially reduced radiation; intuitive interface.	Line‐of‐sight issues; registration accuracy; hardware ergonomics.	Spine/pelvic trauma, arthroplasty.
Custom implants	Implant manufactured based on patient's specific anatomy from CT/magnetic resonance imaging.	Perfect anatomical fit; addresses severe bone defects; optimised load distribution.	High cost; long manufacturing lead time; complex design process.	Musculoskeletal oncology, complex revision arthroplasty.

*Note*: This table summarises the core principles, key benefits and limitations and common clinical applications for the main technologies used for intraoperative execution.

### Robotic‐assisted surgery and navigation

Robotic‐assisted systems use a preoperative plan and real‐time tracking to guide bone preparation with high accuracy [3].

- Precision gains: These systems achieve submillimetre and subdegree accuracy, with studies reporting significantly lower rates of alignment outliers (>3° deviation) in TKA compared to manual techniques [11, 52].
- Clinical relevance: This precision enables reliable execution of personalised alignment plans and facilitates dynamic ligament balancing, potentially reducing soft tissue releases [29]. In THA, it improves acetabular component placement for patients with abnormal spinopelvic motion, which is critical for reducing dislocation risk [28, 47].

### PSI and custom implants

PSI uses preoperative imaging to create 3D‐printed cutting guides matched to a patient's anatomy [5].

- Precision gains: PSI improves the accuracy and reproducibility of achieving the planned coronal alignment in TKA compared to conventional instruments [12, 33, 51].
- Clinical relevance: By improving alignment and component fit, PSI can reduce operative time and blood loss [33]. Custom implants are most relevant for severe bone deformity or large defects in oncology and revision surgery, where standard implants are inadequate [6, 7].

### Augmented reality (AR) in orthopaedic surgery

AR overlays digital information (e.g., surgical guides) onto the surgeon's real‐world view [13].

- Precision gains: In spinal trauma, AR‐guided pedicle screw placement has shown accuracy comparable or superior to navigation [27, 50]. Early TKA data suggests similar alignment accuracy to other computer‐assisted methods [9], while use in shoulder arthroplasty has shown promise [30].
- Clinical relevance: AR enhances spatial awareness and can significantly reduce fluoroscopy time and radiation exposure for the patient and surgical team in trauma and spine surgery [50].

### Postoperative management and broader applications

Precision orthopaedics extends beyond the operating room, influencing diagnostics, rehabilitation and management of complex pathologies.

### Molecular diagnostics and biomarkers

Molecular and genetic diagnostics provide biological precision, moving care from reactive to predictive. Key applications are summarised in Table 2. In diagnosing periprosthetic joint infection (PJI), synovial fluid alpha‐defensin and serum markers like IL‐6 offer high accuracy, helping distinguish septic from aseptic failure [2, 53]. Next‐generation sequencing (NGS) further enhances this by enabling culture‐independent detection of pathogens [25] and is also integral to tumour profiling in musculoskeletal oncology [1]. Beyond infection, genetic profiling (e.g., HLA‐B27) aids in risk stratification for inflammatory conditions [32], while researchers are exploring markers to predict the success of regenerative medicine procedures like cartilage repair.

**Table 2:** Summary of key biomarkers in orthopaedic precision medicine.

Category	Example Biomarkers	Clinical Application
Periprosthetic joint infection (PJI)	Alpha‐defensin (synovial), interleukin‐6 (IL‐6), C‐reactive protein (CRP), D‐dimer (serum).	High‐accuracy diagnosis of PJI, distinguishing infection from aseptic loosening.
Inflammatory arthropathies	Human leucocyte antigen b27 (HLA‐B27), rheumatoid factor (RF), anti‐CCP.	Diagnosis and risk stratification for conditions like ankylosing spondylitis and rheumatoid arthritis.
Musculoskeletal oncology	Gene mutations (e.g., MDM2), RNA expression profiles.	Diagnosis, prognosis and selection of targeted therapies for sarcomas.
Regenerative medicine	Growth factors (e.g., BMPs), mesenchymal stem cell markers (e.g., CD105).	Guiding and monitoring response to cell‐based therapies for cartilage or bone repair.

*Note*: This table provides an overview of important biomarkers categorised by their clinical application, including PJI, inflammatory conditions and regenerative medicine.

### Functional assessment and personalised rehabilitation

Objective functional data is critical for tailoring recovery. 3D gait analysis and wearable inertial measurement units (IMUs) provide quantitative data on joint kinematics, identifying subtle asymmetries that can guide targeted physical therapy [15, 46]. This approach is used not only in lower limb arthroplasty but also to evaluate outcomes in spinal deformity correction [4] and foot and ankle surgery [38].

This data feeds into personalised rehabilitation programs delivered via digital therapeutic platforms. These apps use sensor feedback or smartphone cameras to guide exercises and adapt programs based on a patient's real‐time progress, improving engagement and supporting remote monitoring [8, 39]. Such individualised regimens have also shown promise in improving return‐to‐function timelines in sports medicine [54].

### Adoption, barriers and future directions

Despite clear technological advantages, the adoption of precision orthopaedics is not uniform. These tools are most commonly used in high‐volume academic centres, while barriers slow widespread implementation.

- Cost: Robotic systems and custom implants carry a significant financial investment, which can be prohibitive for smaller hospitals and in healthcare systems with strict cost controls [35].
- Learning curve: Adopting new technologies like robotics or AR requires substantial training and can initially increase operative times during the early phase of adoption [35].
- Evidence gaps: While improvements in accuracy are well‐documented, robust, long‐term data demonstrating superiority in implant survival and cost‐effectiveness are still maturing for some technologies.
- Logistics and workflow: Integrating advanced imaging, planning software and new intraoperative equipment requires significant changes to established clinical workflows.

The future lies in overcoming these barriers. As technology matures, costs are expected to decrease, and AI will continue to streamline planning and data analysis. The accumulation of long‐term data from registries will clarify the indications and cost‐effectiveness of each technology, ultimately defining a new standard of personalised orthopaedic care.

## CONCLUSION

Precision medicine in orthopaedics represents a clear shift from a standardised to an individualised model of care. From AI‐driven diagnostics and patient‐specific surgical planning to robotic execution and personalised rehabilitation, these tools offer the potential to improve accuracy, optimise function and reduce complications. While many technologies are already in clinical use, challenges in cost, training and the need for long‐term outcome validation remain significant barriers to universal adoption. The overarching goal, however, remains constant: to deliver the right intervention, for the right patient, at the right time—anchored in robust data, modern tools and sound clinical judgement.

## AUTHOR CONTRIBUTIONS

**Patrick Sadoghi**: Supervision; conceptualisation; data curation; writing—review and editing. **Amir Koutp**: Investigation; methodology; writing—review and editing, contributed equally to first author. **Elmar Herbst**: Supervision; data curation; review and editing. **Giuseppe Milano**: Supervision; data curation; review and editing. **Volker Musah**: Supervision; data curation; review and editing. **Michael T. Hirschmann**: Conceptualisation; data curation; review and editing.

## CONFLICT OF INTEREST STATEMENT

Michael T. Hirschmann: Consulting fees from Depuy Synthes and Symbios; Honoraria for Lectures and Support attending Meetings from Depuy Synthes, Symbios, and S&N; Participation on Advisory Board for Depuy Synthes; Leadership positions in KSSTA Journal, ESSKA, German Knee Society and Personalised Arthroplasty Society. Patrick Sadoghi: Industry grants from DePuy Synthes, Johnson & Johnson, alphamed, and Medacta; Editorial Board Member for JOA, KSSTA and Arthroscopy. Giuseppe Milano: ESSKA Journals Deputy EiC or Associate Editor, Medacta: Research support; Arthrex: Research support, FGP: Research support; Medics: Research grant, Rotator Cuff Study Group: Chairman, Arthroscopy: Emeritus Associate Editor, Minerva Orthopaedics: Associate Editor; Joints: Funding Editor. Elmar Herbst: Deputy Editor‐in‐Chief of Knee Surgery, Sports Traumatology & Arthroscopy (KSSTA). Volker Musah: Grants: NIH, DOD, Consulting fees: NewClip, Smith & Nephew, Patents: Ipad Technology, ACL Study Group, Stock: Ostesys. The remaining author declares no conflicts of interest.

## ETHICS STATEMENT

The authors have nothing to report.

## Data Availability

Data supporting the findings of this study are available upon reasonable request from the corresponding author.
